# Sick for science: experimental endotoxemia as a translational tool to develop and test new therapies for inflammation-associated depression

**DOI:** 10.1038/s41380-020-00869-2

**Published:** 2020-09-01

**Authors:** Julie Lasselin, Mats Lekander, Sven Benson, Manfred Schedlowski, Harald Engler

**Affiliations:** 1 Institute of Medical Psychology and Behavioral Immunobiology, University Hospital Essen, University of Duisburg-Essen, Hufelandstrasse 55, 45122 Essen, Germany; 2 Stress Research Institute, Stockholm University, 10691 Stockholm, Sweden; 3 Department of Clinical Neuroscience, Division for Psychology, Karolinska Institutet, Nobels väg 9, 17177 Stockholm, Sweden; 4 Osher Center for Integrative Medicine, ME Neuroradiologi, Karolinska Universitetssjukhuset, Stockholm, Sweden

**Keywords:** Depression, Biological techniques, Neuroscience

## Abstract

Depression is one of the global leading causes of disability, but treatments remain limited and classical antidepressants were found to be ineffective in a substantial proportion of patients. Thus, novel effective therapies for the treatment of depression are urgently needed. Given the emerging role of inflammation in the etiology and pathophysiology of affective disorders, we herein illustrate how experimental endotoxemia, a translational model of systemic inflammation, could be used as a tool to develop and test new therapeutic options against depression. Our concept is based on the striking overlap of inflammatory, neural, and affective characteristics in patients with inflammation-associated depression and in endotoxin-challenged healthy subjects. Experimental administration of endotoxin in healthy volunteers is safe, well-tolerated, and without known long-term health risks. It offers a highly standardized translational approach to characterize potential targets of therapies against inflammation-associated depression, as well as to identify characteristics of patients that would benefit from these interventions, and, therefore, could contribute to improve personalization of treatment and to increase the overall rate of responders.

## Introduction

Depression is a highly prevalent mental disorder and one of the leading causes of disability worldwide. Globally, an estimated 322 million people are affected by depression [1]. Although effective therapies are available, about one-third of patients with depression fail to respond to treatment with classical first-line antidepressants, such as selective serotonin reuptake inhibitors (SSRIs) [2]. Consequently, there is a pressing need to identify new targets for the development of tailored therapies for those patients who exhibit resistance to the existing treatments.

During the last decade, an extensive body of experimental and clinical evidence has accumulated demonstrating that inflammation is an important factor in the etiology and pathophysiology of major depressive disorder (MDD), at least in a subgroup of patients [3–7]. Consistent with a view of depression as a multifactorial condition, it is thus now well accepted that inflammation contributes to so-called “inflammation-associated depression” [8, 9]. General population studies demonstrate that about 30% of the individuals who were taking antidepressants or who were hospitalized for depression had increased levels (i.e., >3 mg/L) of C-reactive protein (CRP), a clinical marker of inflammation [10, 11]. This subgroup of patients also exhibits increased systemic levels of pro-inflammatory cytokines compared to healthy individuals [12]. Given that patients suffering from inflammation-associated depression typically show resistance to classic antidepressants such as SSRIs [13, 14], this sub-population of patients represents a particular challenge to treat.

Herein, we propose to take advantage of experimental endotoxemia, a well-characterized model of experimental systemic inflammation, to support the development of therapies for this subgroup of patients. Employing this model in healthy volunteers has already provided valuable insights into the mechanisms underlying inflammation-associated depression and could, in the next step, be used to identify new therapeutic targets and to test new treatment strategies that are specifically directed against inflammation-associated depression.

## Endotoxin-induced inflammation: effects on mood and behavior

**Fig. 1:**
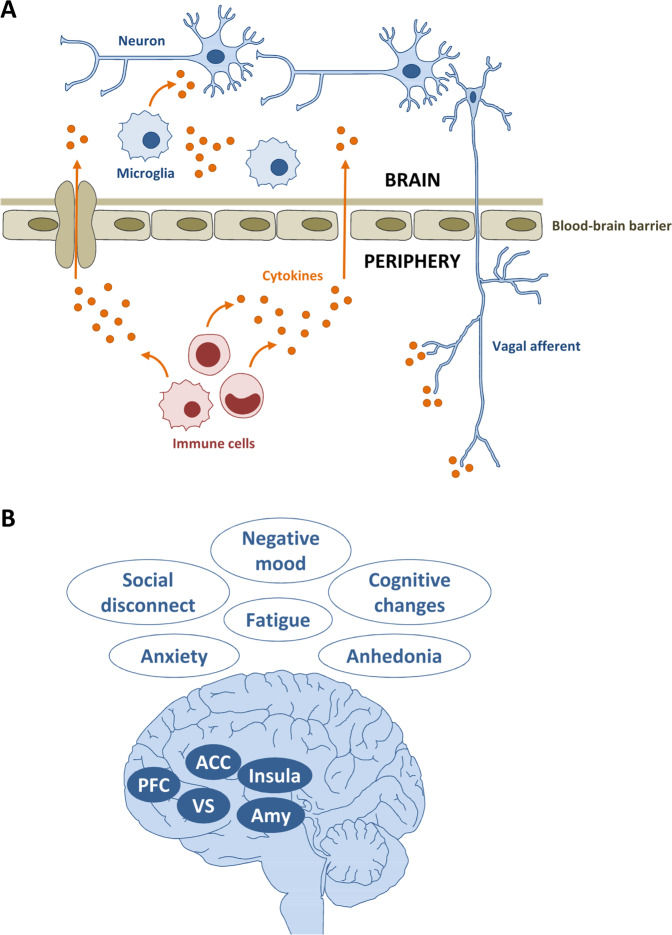
Immune-to-brain communication pathways and cytokine-induced brain and behavioral changes. **a** Immune cells produce cytokines that circulate in the blood and signal the brain via multiple afferent pathways that can act in parallel. These routes comprise activation of vagal sensory neurons projecting to the brainstem, active transport of cytokines across the blood-brain barrier (BBB) via cytokine-specific saturable transporters, and passive diffusion of cytokines in brain areas with incomplete BBB (e.g., circumventricular organs and choroid plexus). The peripheral cytokine signal also activates microglia, which can, in turn, produce cytokines. **b** Cytokine signals reaching the brain trigger functional changes in brain areas involved in emotion processing and mood regulation, such as the insula, amygdala (Amy), ventral striatum (VS), anterior cingulate cortex (ACC), and prefrontal cortex (PFC). This leads to the behavioral reorganization of the individual and to affective changes. The figure was generated using images purchased from Motifolio Inc. (Ellicott City, MD, USA).

Systemic inflammation can be experimentally elicited in both animals and healthy humans by administration of purified bacterial endotoxin (lipopolysaccharide [LPS]). LPS is a cell-wall component of Gram-negative bacteria and a prototypical pathogen-associated molecular pattern that activates the innate immune system through a Toll-like receptor 4-dependent pathway [15]. Intravenous endotoxin injection to healthy humans rapidly triggers a well-described inflammatory cascade, with increased blood and cerebrospinal fluid (CSF) concentrations of cytokines and acute phase proteins [16]. Importantly, a similar signature of inflammatory changes, including elevated blood levels of interleukin (IL)-6, tumor necrosis factor (TNF)-α, and CRP, was also found in inflammation-associated depression [12, 17, 18]. Experimental studies in animals have identified several pathways by which peripheral cytokines can propagate their signal to the brain to induce behavioral and mood changes (Fig. 1a, see ref. [19] for review). Engagement of these afferent pathways triggers inflammatory changes within the brain, affecting neural activity and neurotransmitter release in brain areas involved in emotion processing and mood regulation, such as limbic and cortical regions (Fig. 1b, see refs. [20–23] for reviews). This ultimately leads to the behavioral reorganization of the sick individual, who exhibits reduced locomotion, food consumption, and social exploration, commonly referred to as “sickness behavior” [24]. Interestingly, sick animals also exhibit depressive-like behaviors, such as increased immobility in the forced swim and the tail suspension tests [25], as well as reduced incentive motivation [26].

In humans, the endotoxin-induced inflammatory response is accompanied by negative affective and behavioral changes that resemble core symptoms of depression. Within two hours, individuals challenged with endotoxin typically develop depressive symptoms and negative mood, characterized by an increase in sadness, lassitude, anhedonia, and anxiety, which last for about 4–5 h [16, 27–31]. LPS-treated individuals additionally feel tired and sleepy, have reduced appetite, and engage less in social interactions [32–37]. Experimental endotoxemia also affects incentive motivation [38, 39], and alters the cognitive processing of negative information [40]. The magnitude of the inflammatory response in the circulation and CSF has been found to correlate with these affective changes [16, 27, 28, 34, 38]. Since LPS induces a well-described cascade of inflammatory changes together with behavioral and affective symptoms that are highly relevant for depression, experimental endotoxemia represents a unique model to investigate the role of inflammation in core symptoms of depression and their underlying mechanisms in humans [23, 41, 42].

Interestingly, such as with depression [43], there is some evidence for sex differences in the inflammatory and behavioral responses to experimental endotoxemia [44], but so far the findings are inconsistent. Some studies found a heighted pro-inflammatory response and more pronounced mood disturbances after LPS administration in women compared to men, while others did not [45–50]. It is possible that this heterogeneity is related to the hormonal status of the female volunteers, but this needs to be confirmed in future studies.

## Endotoxin-induced inflammation and neuroimaging findings

Several studies have used experimental endotoxemia in healthy human volunteers to investigate the brain mechanisms underlying inflammation-induced behavioral and mood changes relevant for depression [22, 51]. These studies have analyzed, for example, alterations in the neural processing of social stimuli or rewards [30, 52–55], brain functional connectivity, glucose metabolism, and activation of glial cells [32, 33, 56, 57].

Regarding social and emotional functioning, a study investigated the neural correlates of inflammation-induced social disconnection and increased emotional responsiveness, and could show that the LPS-induced rise in circulating IL-6 levels was significantly positively correlated with the activity in the dorsal anterior cingulate cortex (dACC) and anterior insula during a social exclusion task in females (but not males) [53]. Furthermore, negative social feedback after endotoxin injection led to more pronounced BOLD responses in the amygdala, dACC, and the dorsomedial prefrontal cortex, although these changes were not reflected in behavioral measures [58]. On a related note, a functional magnetic resonance imaging (fMRI) study addressing neural responses to emotional stimuli during endotoxin-induced immune activation revealed increased activation of prefrontal regions, i.e., the inferior orbitofrontal, medial and superior prefrontal cortices, which are closely connected to the amygdala [55]. In another endotoxin study, increased amygdala responses were found in response to socially threatening stimuli such as fearful faces, with amygdala responses being related to feelings of social disconnection [59]. Altogether, endotoxin-induced inflammation seems to increase neural responses in the amygdala, ACC, and prefrontal regions during the processing of social and emotional information. These brain regions are—beside other functions—key contributors to emotion processing and regulation, and structural and functional alterations in these regions have been implicated in depression pathology. Indeed, amygdala responses to negative stimuli are more pronounced and longer lasting in depressed patients [60], which could be at least partially explained by altered inhibitory control from prefrontal regions [61]. In addition, increased ACC activation to emotional stimuli has been reported in depression [22, 62].

Furthermore, significant reductions in ventral striatal activity to reward cues have been found during experimental endotoxemia [30], indicating that endotoxin-induced inflammatory activation alters the brain’s sensitivity to rewards [30]. The clinical validity of such findings is indirectly supported by the observation that increased CRP levels were associated with reduced corticostriatal connectivity in patients with MDD [63]. In depression, increased sensitivity to negative feedback has been repeatedly observed [22], but its potential relationship to inflammation has so far only been experimentally explored in a model of typhoid vaccination, showing a shift in relative sensitivity to punishment as compared to reward [64]. If such effects could be also induced by endotoxin administration, this opens up possibilities to manipulate a core feature of depression and to use neuroimaging to study its neural signatures. As early changes in reward sensitivity predict treatment outcomes [65], and because inflammation is indicative of treatment-resistant depression [14], a model to better understand this shift would be valuable.

Recently, a number of studies have shown that experimental inflammation results in rapid modulation of interoceptive pathways, including the insular and cingulate cortices [56, 57, 66, 67]. Importantly, altered functioning in these pathways is also a central feature in major depression [68, 69], and is believed to represent neural substrates of psychological states common to depression and sickness, such as fatigue, malaise, and social disconnect [70]. These fMRI findings were supported by the results of a positron emission tomography (PET) study, in which changes in glucose metabolism in the insula (and to some extent also in the cingulate) were correlated with increased depressive symptoms after endotoxin injection [33].

PET imaging also has recently been used to measure microglia activity by selectively targeting the 18 kDa mitochondrial translocator protein (TSPO), which is upregulated in activated microglia. A series of studies have shown increases in TSPO binding after immune provocation and in MDD patients [32, 71–74]. Building on an initial study in baboons [75], a PET study in healthy humans using the radioligand [^11^C]PBR28 observed increased TSPO binding throughout the brain together with more pronounced sickness symptoms after endotoxin injection [32]. This TSPO binding pattern was similar to those in patients with MDD, for which a meta-analysis reported increased TSPO expression in ACC, frontal lobe, prefrontal and temporal cortices, insula, and hippocampus when compared to healthy controls [71]. Interestingly, a recent study showed that higher TPSO values in patients with treatment-resistant depression predicted better treatment response to Celecoxib [76]. However, these findings need to be interpreted with caution, as results of TSPO PET studies in neuroinflammatory conditions or states have shown inconsistent results [77], challenging the general assumption that altered TSPO expression or binding unequivocally mirrors neuroinflammation [78, 79]. In line with this, no changes in TSPO binding were found after IFN-α immune challenge in healthy human volunteers [80], or in patients with rheumatoid arthritis [81], or severe seasonal allergy [82].

Taken together, neuroimaging techniques can provide important insights into the neural pathways and physiological processes involved in behavioral and mood-related symptoms during endotoxin-induced inflammation. The above findings suggest that these symptoms are at least partially mediated by functional changes in subcortical and prefrontal brain regions. Importantly, the activation of these regions show a striking overlap to neural changes found in inflammation-associated depression, suggesting that the chronic alteration of these pathways by inflammation leads to the development of clinical depression [22, 51]. While there is some support for specific neural substrates of inflammation-associated depression, including alterations of ACC activity while processing negative affective stimuli, and inhibited activity of the ventral striatum during reward tasks (as outlined in ref. [83]), future imaging studies implementing identical experimental tasks and techniques to compare behavioral and brain responses between healthy endotoxin-treated individuals, and patients with inflammation-associated and “typical” depression are suggested.

## Experimental endotoxemia as a tool to develop and test therapies for inflammation-associated depression

**Fig. 2:**
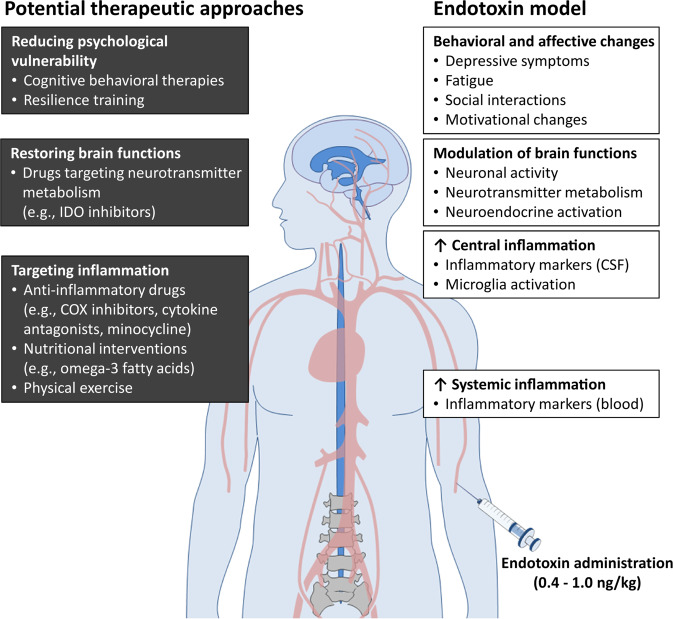
Potential treatment targets and therapeutic approaches that can be studied and tested using human experimental endotoxemia. Administration of endotoxin leads to a cascade of inflammatory, neural, and affective changes that are highly relevant for depression and that can be measured and treated at different levels. Potential treatment options include pharmacological and non-pharmacological therapies targeting the peripheral and central inflammatory responses, neurotransmitter metabolism, or the behavioral/psychological vulnerability. The figure was generated using images purchased from Motifolio Inc. (Ellicott City, MD, USA).

Thus far, experimental endotoxemia has been used in animals and humans to mainly identify the afferent (immune-to-brain) communication pathways and to characterize inflammation-induced behavioral and neural changes. Given the overlap of inflammatory, neural, and affective characteristics in patients with inflammation-associated depression and in endotoxin-challenged healthy subjects, we herein emphasize that experimental endotoxemia constitutes a useful translational model for the development of therapies for inflammation-associated depression (Fig. 2), as previously suggested for inflammatory diseases [84]. Furthermore, we propose that this model could also serve as a tool to define the characteristics of populations of patients that would benefit most from therapies targeting inflammation-associated depression.

Ever since the role of inflammation in the pathophysiology of depression gained interest, various potential pharmacological targets have been identified for the treatment of inflammation-associated depression (see refs. [4, 85, 86] for reviews). Such targets include molecules involved in immune-to-brain communication such as pro-inflammatory cytokines and prostaglandins [87]. In this regard, the prostaglandin synthesis inhibitors celecoxib (cyclooxygenase [COX]-2 inhibitor) and aspirin (COX-1 and COX-2 inhibitor) have been found to enhance the therapeutic efficacy of classical antidepressants [88, 89]. Furthermore, depressed patients with signs of inflammation showed better improvement in depressive symptoms when treated with selective cytokine antagonists, such as the TNF-inhibitors etanercept and infliximab, compared to patients receiving placebo [90, 91]. Two meta-analyses also indicate potential beneficial effects of anti-inflammatory treatments on depressive symptoms [92, 93], although the number of studies included in these meta-analyses was limited (less than 20). Other studies have suggested to directly targeting neuroinflammatory processes by drugs that inhibit microglia activation such as the tetracycline antibiotic minocycline [5, 94, 95].

Alternative potential targets for therapies against inflammation-associated depression could be neurotransmitter metabolism pathways that are demonstrably affected by inflammation, as extensively reviewed previously [3, 96]. For instance, activation of the enzyme indoleamine 2,3-dioxygenase (IDO), which degrades tryptophan into kynurenine (KYN) to the detriment of serotonin synthesis, appears to be one key mechanism for inflammation-induced changes in depressive symptoms [21]. Activated microglia converts kynurenine (KYN) into quinolinic acid (QUIN), a highly potent N-methyl-D-aspartate (NMDA) receptor agonist that triggers the release of the excitatory neurotransmitter glutamate. Increased brain glutamate, notably in the basal ganglia and dACC, has recently been shown in inflammation-associated depression [97]. The current use of low-dose ketamine as antidepressant therapy in patients with treatment-resistant depression is based on ketamine-blocking effects on NMDA-mediated glutamate transmission. Interestingly, particularly patients with elevated levels of inflammatory markers seem to benefit from low-dose ketamine treatment [98, 99], which is in line with the findings from rodent studies showing that ketamine prevents LPS-induced depressive-like behavior [100–102].

Potential therapies for inflammation-associated depression are not restricted to pharmacological therapies. Nutritional interventions such as supplementation with anti-inflammatory omega-3 polyunsaturated fatty acids can improve inflammatory states/conditions [103], or favor a good gut microbiota balance [104]. This, in turn, is likely to modulate mood symptoms [105, 106]. In the same way, exercise interventions have anti-inflammatory effects [107] and could, thus, improve inflammation-associated depressive symptoms [108]. Furthermore, non-pharmacological therapies can target the behavioral changes associated with inflammation rather than inflammation per se. Such therapies would focus on improving psychological factors that could confer vulnerability to inflammation-induced mood changes, such as sleep disturbances [46], pre-existent anxiety [31] and depressive symptoms [109], trait sensitivity to social disconnection [110], and negative affectivity [111]. Importantly, psychological and mind-body therapies appear to also have positive impact on the inflammatory state [112, 113], and therefore arise as an interesting tool for inflammation-associated depression.

Altogether, the challenge to treat inflammation-associated depression does not seem to come from a lack of possible targets (Fig. 2), but rather from the difficulty to determine the efficacy of such therapies. In particular, clinical trials are highly challenging with respect to time, achievability, as well as financing. Furthermore, clinical trials addressing inflammation-associated depression might lead to false-negative results, as only a sub-population of depressed patients would benefit from these therapies, and positive effects might be diluted in the overall group of depressed patients. Thus, we believe that experimental endotoxemia could be an important tool to countervail these issues. The model might be particularly useful in the preclinical phase to test whether a particular drug/treatment is effective in preventing affective and behavioral changes in endotoxin-challenged healthy humans. This would nicely complement animal studies (e.g., [100–102, 114–116]), by developing tailored therapies for patients with inflammation-associated depression, and testing them in a human population free of comorbidities and in a limited period of time.

Two previous studies have tested whether a pre-treatment with an SSRI or with bupropion prevents mood alterations induced by endotoxin [29, 117], with limited effects, in line with the notion that inflammation-associated depression is resistant to classic antidepressant therapies [13]. Future studies will need to investigate the potential usefulness of the therapies described above against inflammation-associated depression.

## Characterization of the patient target population

Although the above therapeutic options might be to some extent promising for the treatment of inflammation-associated depression, the success of such therapies largely depends on the proper identification of patients that benefit from this kind of interventions. One of the biomarkers that have gained strong interest in the quest for selecting depressed patients with inflammation is CRP [118]. Measuring circulating CRP concentrations has already been suggested as a marker to identify depressed patients that would benefit most from anti-inflammatory treatments [91]. A major advantage of CRP is that it is highly standardized and relatively easy to measure in the blood (when following recommendations [119]). However, CRP has two isoforms, one that is not soluble in plasma, with pro-inflammatory properties (mCRP), and one highly soluble and possessing *anti*-inflammatory properties (pCRP). The standard CRP assays measure both CRP isoforms [120]. Consequently, although clinically high levels of CRP (>10 mg/L) probably relate to increased production of mCRP, it is less clear when only a slight elevation in CRP is observed [120]. Thus, it remains unclear whether moderate increases in CRP levels can indicate with confidence a risk for inflammation-associated depression.

Experimental endotoxemia could help identifying the characteristics of patients that would benefit from therapies targeting inflammation-associated depression. Arguably, these patients would exhibit an increased inflammatory state, but also higher target cell sensitivity to the effects of cytokines, and thus would show a stronger behavioral response to endotoxin [44, 121]. Using experimental models of inflammation, recent studies have provided clues regarding variables possibly associated with higher emotional and behavioral response to cytokines, such as baseline psychological state (e.g., state anxiety, negative affectivity, perceived stress) [31, 40, 111, 121], sleep disturbances [46], and baseline activity of some transcription factors [122]. Identifying further the characteristics that predict a higher and/or prolonged emotional response to inflammation would help determining which subgroup of patients would benefit the most from therapies for inflammation-associated depression.

## Demarcation from other models of inflammation-associated depression

In addition to experimental endotoxemia, two other models have provided valuable insights into inflammation-associated depression: the IFN-α model and the typhoid vaccination model. IFN-α treatment has been clinically used, and about half of the patients develop depressive symptoms within 8–12 weeks after the onset of IFN-α treatment [123]. Studies in IFN-α-treated patients have greatly expanded knowledge on the role of inflammation in depression [96]. Interestingly, chronic IFN-α treatment in hepatitis C patients [124] induced a comparable pattern of CSF cytokine changes as in endotoxin-treated healthy subjects [16]. Moreover, both acute LPS administration and chronic IFN-α administration induced similar changes in brain function, including reduced activation of ventral striatum in response to reward [125], increased glutamate in the ACC [126], and reduced functional connectivity [127]. In addition, changes in basal ganglia were observed acutely (4 h) after the administration of IFN-α, and predicted the long-term development of fatigue [128, 129]. Although the model of chronic IFN-α administration allows assessing the long-term effects of inflammatory activation, IFN-α cannot be applied chronically to healthy volunteers for ethical reasons.

Administration of typhoid vaccine can be safely used in healthy subjects, and triggers a mild immune activation and very subtle changes in mood in a small proportion of subjects [130, 131]. Changes in brain functions have been also observed after typhoid vaccination, such as increased activation of the ACC during emotional face processing [130], increased activity in the amygdala and in the insula [132], and reduced activity in the ventral striatum in response to reward [64], in line with those observed during experimental endotoxemia. However, because of the response being very mild and not as reproducible as in experimental endotoxemia, this model seems less useful as a tool for the development and testing of new treatments against inflammation-associated depression.

## Advantages and limitations of the experimental endotoxemia model

The model of experimental endotoxemia offers many advantages. First, because of the transient nature of the endotoxin-induced inflammatory and behavioral responses, the efficacy of a potential therapeutic intervention can be assessed within a relatively short period of time. Second, dose-effect relationships can be easily obtained by modulating the dose of endotoxin [28, 133, 134]. For example, a dose of 0.4 ng/kg body weight (bw) induces very slight behavioral changes that are imperceptible to participants, while a dose of 2.0 ng/kg bw triggers very strong sickness symptoms and emotional distress in the majority of participants. Note that the majority of studies assessing LPS-induced changes in mood and negative affect used doses between 0.4–1.0 ng/kg bw, while higher doses (2.0–4.0 ng/kg bw) are mainly used in studies focusing on sepsis-related symptoms. Third, since the response to LPS is highly conserved across vertebrate species, the model allows forward and reverse translation of the findings from animals to humans [135]. Using experimental endotoxemia in humans additionally provides an evident benefit of assessing feeling states, which are not necessarily reflected in objective behavioral changes (e.g., a feeling of fatigue does not necessarily translate into lower physical activities) [135]. Even though this model is not a model of depression per se (see below), the model of experimental endotoxemia provides crucial information about inflammation-induced affective changes and the underlying mechanisms.

Some limitations of the human experimental endotoxemia model as a model of depression need to be considered as well. The main limitation lies in the discrepancy of the severity and chronicity of the LPS-induced emotional symptoms compared to the symptoms of MDD. While emotional symptoms in MDD last for at least two weeks, the behavioral and emotional changes induced by endotoxin are acute and subside completely 6–8 h after endotoxin injection. Repeated or chronic administration of very low doses of endotoxin have been attempted to extend the LPS-induced behavioral and physiological symptoms [136], but these approaches were hampered by endotoxin tolerance. One critical question is whether the acute changes observed during experimental endotoxemia would turn into clinical depression if inflammation and activation of the immune-to-brain pathways would persist for a longer period of time. Despite similarities in the emotional and neural changes observed under acute experimental inflammation and in inflammation-associated depression [22, 23, 41, 42, 51], one can only speculate if such changes would become chronic with ongoing inflammation, as the acute LPS-induced mood effects last only for a few hours. However, findings from IFN-α treatment studies suggest that this indeed might be the case, by demonstrating that acute IFN-α-induced changes predicted the later development of neuropsychiatric symptoms [128, 129, 137]. An important research question to investigate would be to which extent the emotional and neural responses to LPS predict the later development of depression. In any case, phase-I studies to determine the potential usefulness and safety of a therapy do not require having a model that reflects precisely the disease. Given the characteristics and advantages of the model of experimental endotoxemia described above, phase-I studies of therapies against inflammation-associated depression would benefit from using this model.

Another limitation is that experimental endotoxemia has only been used in very healthy subjects, without physiological diseases, medications, or mood, sleep, and stress disorders, apart from one recent study in obese but metabolically healthy individuals [138]. Thus, endotoxin-induced neural and behavioral changes in populations that are at higher risk at developing depression, for instance individuals suffering from diabetes, chronic pain, or cancer [139], and in depressed individuals, remain unknown and need to be investigated.

## Methodological considerations

Although experimental endotoxemia is a safe model of systemic inflammation when performed under controlled conditions, some technical recommendations need to be considered. As endotoxin-induced systemic inflammation leads to increases in heart rate and body temperature, and can (depending on the dose) induce nausea, headache, as well as dizziness and acute drop in blood pressure, medical supervision is necessary. Furthermore, only low doses of LPS can be used when studying neural mechanisms using brain imaging, to limit shivering and nausea in the scanner. In addition, individuals should be monitored until the sickness response has subsided (i.e., until 6–8 h after endotoxin injection) before being discharged after a medical examination to insure their safety.

Various research groups have used the model of experimental endotoxemia in humans, with variations in experimental procedures. Although it is unclear how each parameter of the procedure affects the response to endotoxin, some parameters are likely to modulate the immune and/or brain outcomes, such as time of injection [140], individuals’ expectations [134], and fasting state [141]. Furthermore, various tools have been used to measure the depressive response, including semi-structured interviews (e.g., Montgomery-Åsberg Depression Inventory MADRS) and self-administered questionnaires (e.g., Profile of Mood States POMS). Lassitude and fatigue have rarely been assessed specifically [142], but rather as subscale from the MADRS or POMS. State anxiety seems as the only symptom that was measured across studies consistently with the State-Trait Anxiety Scale (STAI). While it is difficult to advise on the choice of specific scales, we recommend to compile scales designed to assess specific symptoms, to have an overview of the inflammation-induced specific neuropsychiatric changes and how they relate to a general state of sickness [143]. Furthermore, it can be useful to assess objective behavioral aspects, such as objective motivational [38, 39] and appetite [37] changes, as they provide additional information on the specific mechanisms underlying the development of affective symptoms [135]. Such procedures might overcome the limitation of sickness as a generalized behavioral response to the inflammatory stimulus, and increase precision and specificity for depression-relevant behavioral changes. We also urge to report protocol details when using human experimental endotoxemia, and call for a standardization of the procedure to make the best use of this model for the proposed purpose.

## Conclusion

Experimental administration of endotoxin to healthy volunteers offers a highly standardized translational model of systemic inflammation that has been successfully used to investigate the mechanisms underlying inflammation-associated behavioral and mood changes. It has been proven to be safe, well-tolerated, and without any known long-term health risks. The overlap of inflammatory, neural and affective characteristics in endotoxin-challenged healthy subjects and patients suffering from inflammation-associated depression emphasizes that human experimental endotoxemia might serve as a suitable tool in the quest to develop personalized and thereby more effective therapies for major depression.

## References

[1] WHO. Depression and other common mental disorders: global health estimates. WHO; 2017.

[2] Rush AJ, Trivedi MH, Wisniewski SR, Nierenberg AA, Stewart JW, Warden D (2006). Acute and longer-term outcomes in depressed outpatients requiring one or several treatment steps: a STAR*D report. Am J Psychiatry.

[3] Dantzer R, O’Connor JC, Freund GG, Johnson RW, Kelley KW (2008). From inflammation to sickness and depression: when the immune system subjugates the brain. Nat Rev Neurosci.

[4] Miller AH, Raison CL (2016). The role of inflammation in depression: from evolutionary imperative to modern treatment target. Nat Rev Immunol.

[5] Yirmiya R, Rimmerman N, Reshef R (2015). Depression as a microglial disease. Trends Neurosci.

[6] Bell JA, Kivimaki M, Bullmore ET, Steptoe A, Carvalho LA (2017). MRC ImmunoPsychiatry Consortium. Repeated exposure to systemic inflammation and risk of new depressive symptoms among older adults. Transl Psychiatry.

[7] Khandaker GM, Pearson RM, Zammit S, Lewis G, Jones PB (2014). Association of serum interleukin 6 and C-reactive protein in childhood with depression and psychosis in young adult life: a population-based longitudinal study. JAMA Psychiatry.

[8] Dantzer R, O’Connor JC, Lawson MA, Kelley KW (2011). Inflammation-associated depression: from serotonin to kynurenine. Psychoneuroendocrinology.

[9] Lotrich FE (2015). Inflammatory cytokine-associated depression. Brain Res.

[10] Wium-Andersen MK, Orsted DD, Nielsen SF, Nordestgaard BG (2013). Elevated C-reactive protein levels, psychological distress, and depression in 73, 131 individuals. JAMA Psychiatry.

[11] Osimo EF, Cardinal RN, Jones PB, Khandaker GM (2018). Prevalence and correlates of low-grade systemic inflammation in adult psychiatric inpatients: an electronic health record-based study. Psychoneuroendocrinology.

[12] Dowlati Y, Herrmann N, Swardfager W, Liu H, Sham L, Reim EK (2010). A meta-analysis of cytokines in major depression. Biol Psychiatry.

[13] Chamberlain SR, Cavanagh J, de Boer P, Mondeli V, Jones DNC, Drevets WC (2019). Treatment-resistant depression and peripheral C-reactive protein. Br J Psychiatry.

[14] Haroon E, Daguanno AW, Woolwine BJ, Goldsmith DR, Baer WM, Wommack EC (2018). Antidepressant treatment resistance is associated with increased inflammatory markers in patients with major depressive disorder. Psychoneuroendocrinology.

[15] Beutler B (2002). TLR4 as the mammalian endotoxin sensor. Curr Top Microbiol.

[16] Engler H, Brendt P, Wischermann J, Wegner A, Rohling R, Schoemberg T (2017). Selective increase of cerebrospinal fluid IL-6 during experimental systemic inflammation in humans: association with depressive symptoms. Mol Psychiatry.

[17] Leighton SP, Nerurkar L, Krishnadas R, Johnman C, Graham GJ, Cavanagh J (2018). Chemokines in depression in health and in inflammatory illness: a systematic review and meta-analysis. Mol Psychiatry.

[18] Felger JC, Haroon E, Patel TA, Goldsmith DR, Wommack EC, Woolwine BJ, et al. What does plasma CRP tell us about peripheral and central inflammation in depression? Mol Psychiatry. 2020;25:1301–11. 10.1038/s41380-018-0096-3 PMC6291384 29895893

[19] D’Mello C, Swain MG (2017). Immune-to-brain communication pathways in inflammation-associated sickness and depression. Curr Top Behav Neurosci.

[20] Felger JC (2017). The role of dopamine in inflammation-associated depression: mechanisms and therapeutic implications. Curr Top Behav Neurosci.

[21] Dantzer R (2017). Role of the kynurenine metabolism pathway in inflammation-induced depression: preclinical approaches. Curr Top Behav Neurosci.

[22] Harrison NA (2017). Brain structures implicated in inflammation-associated depression. Curr Top Behav Neurosci.

[23] Dooley LN, Kuhlman KR, Robles TF, Eisenberger NI, Craske MG, Bower JE (2018). The role of inflammation in core features of depression: Insights from paradigms using exogenously-induced inflammation. Neurosci Biobehav Rev.

[24] Hart BL (1988). Biological basis of the behavior of sick animals. Neurosci Biobehav Rev.

[25] Frenois F, Moreau M, O’Connor J, Lawson M, Micon C, Lestage J (2007). Lipopolysaccharide induces delayed FosB/DeltaFosB immunostaining within the mouse extended amygdala, hippocampus and hypothalamus, that parallel the expression of depressive-like behavior. Psychoneuroendocrinology.

[26] Vichaya EG, Hunt SC, Dantzer R (2014). Lipopolysaccharide reduces incentive motivation while boosting preference for high reward in mice. Neuropsychopharmacology.

[27] Reichenberg A, Yirmiya R, Schuld A, Kraus T, Haack M, Morag A (2001). Cytokine-associated emotional and cognitive disturbances in humans. Arch Gen Psychiatry.

[28] Grigoleit JS, Kullmann JS, Wolf OT, Hammes F, Wegner A, Jablonowski S (2011). Dose-dependent effects of endotoxin on neurobehavioral functions in humans. PLoS ONE.

[29] DellaGioia N, Devine L, Pittman B, Hannestad J (2013). Bupropion pre-treatment of endotoxin-induced depressive symptoms. Brain Behav Immun.

[30] Eisenberger NI, Berkman ET, Inagaki TK, Rameson LT, Mashal NM, Irwin MR (2010). Inflammation-induced anhedonia: endotoxin reduces ventral striatum responses to reward. Biol Psychiatry.

[31] Lasselin J, Elsenbruch S, Lekander M, Axelsson J, Karshikoff B, Grigoleit JS (2016). Mood disturbance during experimental endotoxemia: predictors of state anxiety as a psychological component of sickness behavior. Brain Behav Immun.

[32] Sandiego CM, Gallezot JD, Pittman B, Nabulsi N, Lim K, Lin SF (2015). Imaging robust microglial activation after lipopolysaccharide administration in humans with PET. Proc Natl Acad Sci USA.

[33] Hannestad J, Subramanyam K, Dellagioia N, Planeta-Wilson B, Weinzimmer D, Pittman B (2012). Glucose metabolism in the insula and cingulate is affected by systemic inflammation in humans. J Nucl Med.

[34] Eisenberger NI, Inagaki TK, Mashal NM, Irwin MR (2010). Inflammation and social experience: an inflammatory challenge induces feelings of social disconnection in addition to depressed mood. Brain Behav Immun.

[35] Hermann DM, Mullington J, Hinze-Selch D, Schreiber W, Galanos C, Pollmacher T (1998). Endotoxin-induced changes in sleep and sleepiness during the day. Psychoneuroendocrinology.

[36] Marraffa A, Lekander M, Solsjo P, Olsson MJ, Lasselin J, Axelsson J (2017). Yawning, a thermoregulatory mechanism during fever? A study of yawning frequency and its predictors during experimentally induced sickness. Physiol Behav.

[37] Reichenberg A, Kraus T, Haack M, Schuld A, Pollmacher T, Yirmiya R (2002). Endotoxin-induced changes in food consumption in healthy volunteers are associated with TNF-alpha and IL-6 secretion. Psychoneuroendocrinology.

[38] Lasselin J, Treadway MT, Lacourt TE, Soop A, Olsson MJ, Karshikoff B (2017). Lipopolysaccharide alters motivated behavior in a monetary reward task: a randomized trial. Neuropsychopharmacology.

[39] Draper A, Koch RM, van der Meer JW, Aj Apps M, Pickkers P, Husain M, et al. Effort but not reward sensitivity is altered by acute sickness induced by experimental endotoxemia in humans. Neuropsychopharmacology. 2018;43:1107–18. 10.1038/npp.2017.231 PMC5854801 28948979

[40] Benson S, Brinkhoff A, Lueg L, Roderigo T, Kribben A, Wilde B (2017). Effects of acute systemic inflammation on the interplay between sad mood and affective cognition. Transl Psychiatry.

[41] DellaGioia N, Hannestad J (2010). A critical review of human endotoxin administration as an experimental paradigm of depression. Neurosci Biobehav Rev.

[42] Schedlowski M, Engler H, Grigoleit JS (2014). Endotoxin-induced experimental systemic inflammation in humans: a model to disentangle immune-to-brain communication. Brain Behav Immun.

[43] Altemus M, Sarvaiya N, Neill Epperson C (2014). Sex differences in anxiety and depression clinical perspectives. Front Neuroendocrinol.

[44] Lasselin J, Lekander M, Axelsson J, Karshikoff B (2018). Sex differences in how inflammation affects behavior: What we can learn from experimental inflammatory models in humans. Front Neuroendocrinol.

[45] Engler H, Benson S, Wegner A, Spreitzer I, Schedlowski M, Elsenbruch S (2016). Men and women differ in inflammatory and neuroendocrine responses to endotoxin but not in the severity of sickness symptoms. Brain Behav Immun.

[46] Cho HJ, Eisenberger NI, Olmstead R, Breen EC, Irwin MR (2016). Preexisting mild sleep disturbance as a vulnerability factor for inflammation-induced depressed mood: a human experimental study. Transl Psychiatry.

[47] Karshikoff B, Lekander M, Soop A, Lindstedt F, Ingvar M, Kosek E (2015). Modality and sex differences in pain sensitivity during human endotoxemia. Brain Behav Immun.

[48] Moieni M, Irwin MR, Jevtic I, Olmstead R, Breen EC, Eisenberger NI (2015). Sex differences in depressive and socioemotional responses to an inflammatory challenge: implications for sex differences in depression. Neuropsychopharmacology.

[49] Wegner A, Benson S, Rebernik L, Spreitzer I, Jager M, Schedlowski M (2017). Sex differences in the pro-inflammatory cytokine response to endotoxin unfold in vivo but not ex vivo in healthy humans. Innate Immun.

[50] Moieni M, Tan KM, Inagaki TK, Muscatell KA, Dutcher JM, Jevtic I (2019). Sex differences in the relationship between inflammation and reward sensitivity: a randomized controlled trial of endotoxin. Biol Psychiatry Cogn Neurosci Neuroimaging.

[51] Kraynak TE, Marsland AL, Wager TD, Gianaros PJ. Functional neuroanatomy of peripheral inflammatory physiology: a meta-analysis of human neuroimaging studies. Neurosci Biobehav Rev. 2018;94:76–92. 10.1016/j.neubiorev.2018.07.013 PMC6363360 30067939

[52] Kullmann JS, Grigoleit JS, Wolf OT, Engler H, Oberbeck R, Elsenbruch S (2014). Experimental human endotoxemia enhances brain activity during social cognition. Soc Cogn Affect Neurosci.

[53] Eisenberger NI, Inagaki TK, Rameson LT, Mashal NM, Irwin MR (2009). An fMRI study of cytokine-induced depressed mood and social pain: the role of sex differences. Neuroimage.

[54] Inagaki TK, Muscatell KA, Irwin MR, Moieni M, Dutcher JM, Jevtic I (2015). The role of the ventral striatum in inflammatory-induced approach toward support figures. Brain Behav Immun.

[55] Kullmann JS, Grigoleit JS, Lichte P, Kobbe P, Rosenberger C, Banner C (2013). Neural response to emotional stimuli during experimental human endotoxemia. Hum Brain Mapp.

[56] Labrenz F, Wrede K, Forsting M, Engler H, Schedlowski M, Elsenbruch S (2016). Alterations in functional connectivity of resting state networks during experimental endotoxemia - An exploratory study in healthy men. Brain Behav Immun.

[57] Lekander M, Karshikoff B, Johansson E, Soop A, Fransson P, Lundstrom JN (2016). Intrinsic functional connectivity of insular cortex and symptoms of sickness during acute experimental inflammation. Brain Behav Immun.

[58] Muscatell KA, Moieni M, Inagaki TK, Dutcher JM, Jevtic I, Breen EC (2016). Exposure to an inflammatory challenge enhances neural sensitivity to negative and positive social feedback. Brain Behav Immun.

[59] Inagaki TK, Muscatell KA, Irwin MR, Cole SW, Eisenberger NI (2012). Inflammation selectively enhances amygdala activity to socially threatening images. Neuroimage.

[60] Stuhrmann A, Suslow T, Dannlowski U (2011). Facial emotion processing in major depression: a systematic review of neuroimaging findings. Biol mood Anxiety Disord.

[61] Golkar A, Lonsdorf TB, Olsson A, Lindstrom KM, Berrebi J, Fransson P (2012). Distinct contributions of the dorsolateral prefrontal and orbitofrontal cortex during emotion regulation. PLoS ONE.

[62] Hamilton JP, Etkin A, Furman DJ, Lemus MG, Johnson RF, Gotlib IH (2012). Functional neuroimaging of major depressive disorder: a meta-analysis and new integration of base line activation and neural response data. Am J Psychiatry.

[63] Felger JC, Li Z, Haroon E, Woolwine BJ, Jung MY, Hu X (2016). Inflammation is associated with decreased functional connectivity within corticostriatal reward circuitry in depression. Mol Psychiatry.

[64] Harrison NA, Voon V, Cercignani M, Cooper EA, Pessiglione M, Critchley HD (2016). A neurocomputational account of how inflammation enhances sensitivity to punishments versus rewards. Biol Psychiatry.

[65] Allen TA, Lam RW, Milev R, Rizvi SJ, Frey BN, MacQueen GM (2019). Early change in reward and punishment sensitivity as a predictor of response to antidepressant treatment for major depressive disorder: a CAN-BIND-1 report. Psychol Med.

[66] Benson S, Rebernik L, Wegner A, Kleine-Borgmann J, Engler H, Schlamann M, et al. Neural circuitry mediating inflammation-induced central pain amplification in human experimental endotoxemia. Brain Behav Immun. 2015;48:222–31. 10.1016/j.bbi.2015.03.017 25882910

[67] Karshikoff B, Jensen KB, Kosek E, Kalpouzos G, Soop A, Ingvar M, et al. Why sickness hurts: A central mechanism for pain induced by peripheral inflammation. Brain Behav Immun*.* 2016;57:38–46. 10.1016/j.bbi.2016.04.001 27058164

[68] Mayberg HS, Liotti M, Brannan SK, McGinnis S, Mahurin RK, Jerabek PA (1999). Reciprocal limbic-cortical function and negative mood: converging PET findings in depression and normal sadness. Am J Psychiatry.

[69] Paulus MP, Stein MB (2010). Interoception in anxiety and depression. Brain Struct Funct.

[70] Quadt L, Critchley HD, Garfinkel SN. The neurobiology of interoception in health and disease. Ann N Y Acad Sci. 2018;1428:112–28. 10.1111/nyas.13915 29974959

[71] Enache D, Pariante CM, Mondelli V (2019). Markers of central inflammation in major depressive disorder: A systematic review and meta-analysis of studies examining cerebrospinal fluid, positron emission tomography and post-mortem brain tissue. Brain Behav Immun.

[72] Setiawan E, Wilson AA, Mizrahi R, Rusjan PM, Miler L, Rajkowska G, et al. Role of translocator protein density, a marker of neuroinflammation, in the brain during major depressive episodes. JAMA Psychiatry. 2015;72:268–75. 10.1001/jamapsychiatry.2014.2427 PMC4836849 25629589

[73] Holmes SE, Hinz R, Conen S, Gregory CJ, Matthews JC, Anton-Rodriguez JM (2018). Elevated translocator protein in anterior cingulate in major depression and a role for inflammation in suicidal thinking: a positron emission tomography study. Biol Psychiatry.

[74] Richards EM, Zanotti-Fregonara P, Fujita M, Newman L, Farmer C, Ballard ED, et al. PET radioligand binding to translocator protein (TSPO) is increased in unmedicated depressed subjects. EJNMMI Res. 2018;8:57. 10.1186/s13550-018-0401-9 PMC6029989 29971587

[75] Hannestad J, Gallezot JD, Schafbauer T, Lim K, Kloczynski T, Morris ED (2012). Endotoxin-induced systemic inflammation activates microglia: [(1)(1)C]PBR28 positron emission tomography in nonhuman primates. Neuroimage.

[76] Attwells S, Setiawan E, Rusjan PM, Xu C, Hutton C, Rafiei D, et al. Translocator protein distribution volume predicts reduction of symptoms during open-label trial of celecoxib in major depressive disorder. Biol Psychiatry. 2020;S0006-3223(20)31326-3. 10.1016/j.biopsych.2020.03.007 PMC11878442 32402468

[77] Meyer JH, Cervenka S, Kim MJ, Kresi WC, Henter ID, Innis RB. Neuroinflammation in psychiatric disorders: pet imaging and promising new targets. Lancet Psychiatry. (in press). 10.1016/S2215-0366(20)30255-8 PMC7893630 33098761

[78] Notter T, Coughlin JM, Sawa A, Meyer U (2018). Reconceptualization of translocator protein as a biomarker of neuroinflammation in psychiatry. Mol Psychiatry.

[79] Notter T, Schalbetter SM, Clifton NE, Mattei D, Richetto J, Thomas K, et al. Neuronal activity increases translocator protein (TSPO) levels. Mol Psychiatry. 2020. 10.1038/s41380-020-0745-1. 10.1038/s41380-020-0745-1 PMC8440208 32398717

[80] Nettis MA, Veronese M, Nikkheslat N, Mariani N, Lombardo G, Sforzini L (2020). PET imaging shows no changes in TSPO brain density after IFN-alpha immune challenge in healthy human volunteers. Transl Psychiatry.

[81] Forsberg A, Lampa J, Estelius J, Cervenka S, Farde L, Halldin C (2019). Disease activity in rheumatoid arthritis is inversely related to cerebral TSPO binding assessed by [(11)C]PBR28 positron emission tomography. J Neuroimmunol.

[82] Tamm S, Cervenka S, Forsberg A, Estelius J, Grunewald J, Gyllfors P (2018). Evidence of fatigue, disordered sleep and peripheral inflammation, but not increased brain TSPO expression, in seasonal allergy: a [(11)C]PBR28 PET study. Brain Behav Immun.

[83] Byrne ML, Whittle S, Allen NB (2016). The role of brain structure and function in the association between inflammation and depressive symptoms: a systematic review. Psychosom Med.

[84] Suffredini AF, Noveck RJ (2014). Human endotoxin administration as an experimental model in drug development. Clin Pharm Ther.

[85] Adzic M, Brkic Z, Mitic M, Francija E, Jovicic MJ, Radulovic J (2018). Therapeutic strategies for treatment of inflammation-related depression. Curr Neuropharmacol.

[86] Haroon E, Raison CL, Miller AH (2012). Psychoneuroimmunology meets neuropsychopharmacology: translational implications of the impact of inflammation on behavior. Neuropsychopharmacology.

[87] Kohler O, Krogh J, Mors O, Eriksen Benros M (2016). Inflammation in depression and the potential for anti-inflammatory treatment. Curr Neuropharmacol.

[88] Mendlewicz J, Kriwin P, Oswald P, Souery D, Alboni S, Brunello N (2006). Shortened onset of action of antidepressants in major depression using acetylsalicylic acid augmentation: a pilot open-label study. Int Clin Psychopharmacol.

[89] Muller N, Schwarz MJ, Dehning S, Douhe A, Cerovecki A, Goldstein-Muller B (2006). The cyclooxygenase-2 inhibitor celecoxib has therapeutic effects in major depression: results of a double-blind, randomized, placebo controlled, add-on pilot study to reboxetine. Mol Psychiatry.

[90] Tyring S, Gottlieb A, Papp K, Gordon K, Leonardi C, Wang A (2006). Etanercept and clinical outcomes, fatigue, and depression in psoriasis: double-blind placebo-controlled randomised phase III trial. Lancet.

[91] Raison CL, Rutherford RE, Woolwine BJ, Shuo C, Schettler P, Drake DF (2013). A randomized controlled trial of the tumor necrosis factor antagonist infliximab for treatment-resistant depression: the role of baseline inflammatory biomarkers. JAMA Psychiatry.

[92] Kohler O, Benros ME, Nordentoft M, Farkouh ME, Iyengar RL, Mors O (2014). Effect of anti-inflammatory treatment on depression, depressive symptoms, and adverse effects: a systematic review and meta-analysis of randomized clinical trials. JAMA Psychiatry.

[93] Kappelmann N, Lewis G, Dantzer R, Jones PB, Khandaker GM (2018). Antidepressant activity of anti-cytokine treatment: a systematic review and meta-analysis of clinical trials of chronic inflammatory conditions. Mol Psychiatry.

[94] Husain MI, Chaudhry IB, Husain N, Khoso AB, Rahman RR, Hamirani MM (2017). Minocycline as an adjunct for treatment-resistant depressive symptoms: a pilot randomised placebo-controlled trial. J Psychopharmacol.

[95] Rosenblat JD, McIntyre RS (2018). Efficacy and tolerability of minocycline for depression: a systematic review and meta-analysis of clinical trials. J Affect Disord.

[96] Capuron L, Miller AH (2011). Immune system to brain signaling: neuropsychopharmacological implications. Pharm Ther.

[97] Haroon E, Miller AH (2017). Inflammation effects on brain glutamate in depression: mechanistic considerations and treatment implications. Curr Top Behav Neurosci.

[98] Yang JJ, Wang N, Yang C, Shi JY, Yu HY, Hashimoto K (2015). Serum interleukin-6 is a predictive biomarker for ketamine’s antidepressant effect in treatment-resistant patients with major depression. Biol Psychiatry.

[99] Kiraly DD, Horn SR, Van Dam NT, Costi S, Schwartz J, Kim-Schulze S (2017). Altered peripheral immune profiles in treatment-resistant depression: response to ketamine and prediction of treatment outcome. Transl psychiatry.

[100] O’Connor JC, Lawson MA, Andre C, Moreau M, Lestage J, Castanon N (2009). Lipopolysaccharide-induced depressive-like behavior is mediated by indoleamine 2,3-dioxygenase activation in mice. Mol Psychiatry.

[101] Jeon SA, Lee E, Hwang I, Han B, Park S, Son S (2017). NLRP3 inflammasome contributes to lipopolysaccharide-induced depressive-like behaviors via indoleamine 2,3-dioxygenase induction. Int J Neuropsychopharmacol.

[102] Walker AK, Budac DP, Bisulco S, Lee AW, Smith RA, Beenders B (2013). NMDA receptor blockade by ketamine abrogates lipopolysaccharide-induced depressive-like behavior in C57BL/6J mice. Neuropsychopharmacology.

[103] Ferguson JF, Mulvey CK, Patel PN, Shah RY, Doveikis J, Zhang W (2014). Omega-3 PUFA supplementation and the response to evoked endotoxemia in healthy volunteers. Mol Nutr food Res.

[104] Bruce-Keller AJ, Salbaum JM, Berthoud HR (2018). Harnessing gut microbes for mental health: getting from here to there. Biol Psychiatry.

[105] Larrieu T, Laye S (2018). Food for mood: relevance of nutritional omega-3 fatty acids for depression and anxiety. Front Physiol.

[106] Valles-Colomer M, Falony G, Darzi Y, Tigchelaar EF, Wang J, Tito RY, et al. The neuroactive potential of the human gut microbiota in quality of life and depression. Nat Microbiol. 2019;4:623–32. 10.1038/s41564-018-0337-x 30718848

[107] Starkie R, Ostrowski SR, Jauffred S, Febbraio M, Pedersen BK (2003). Exercise and IL-6 infusion inhibit endotoxin-induced TNF-alpha production in humans. FASEB J.

[108] Paolucci EM, Loukov D, Bowdish DME, Heisz JJ (2018). Exercise reduces depression and inflammation but intensity matters. Biol Psychol.

[109] Benson S, Engler H, Wegner A, Rebernik L, Spreitzer I, Schedlowski M, et al. What makes you feel sick after inflammation? predictors of acute and persisting physical sickness symptoms induced by experimental endotoxemia. Clin Pharmacol Ther. 2017;102:141–51. 10.1002/cpt.618 28074475

[110] Moieni M, Irwin MR, Jevtic I, Breen EC, Cho HJ, Arevalo JM (2015). Trait sensitivity to social disconnection enhances pro-inflammatory responses to a randomized controlled trial of endotoxin. Psychoneuroendocrinology.

[111] Lacourt TE, Houtveen JH, Veldhuijzen van Zanten JJ, Bosch JA, Drayson MT, Van Doornen LJ (2015). Negative affectivity predicts decreased pain tolerance during low-grade inflammation in healthy women. Brain Behav Immun.

[112] O’Toole MS, Bovbjerg DH, Renna ME, Lekander M, Mennin DS, Zachariae R. Effects of psychological interventions on systemic levels of inflammatory biomarkers in humans: a systematic review and meta-analysis. Brain Behav Immun. 2018;74:68–78. 10.1016/j.bbi.2018.04.005 29630988

[113] Morgan N, Irwin MR, Chung M, Wang C (2014). The effects of mind-body therapies on the immune system: meta-analysis. PLoS ONE.

[114] Camara MI, Corrigan F, Jaehne EJ, Jawahar MC, Anscomb H, Baune BT (2014). Effects of centrally administered etanercept on behavior, microglia, and astrocytes in mice following a peripheral immune challenge. Neuropsychopharmacology.

[115] Ohgi Y, Futamura T, Kikuchi T, Hashimoto K (2013). Effects of antidepressants on alternations in serum cytokines and depressive-like behavior in mice after lipopolysaccharide administration. Pharm Biochem Behav.

[116] Demin KA, Sysoev M, Chernysh MV, Savva AK, Koshiba M, Wappler-Guzzetta EA (2019). Animal models of major depressive disorder and the implications for drug discovery and development. Expert Opin Drug Discov.

[117] Hannestad J, DellaGioia N, Ortiz N, Pittman B, Bhagwagar Z (2011). Citalopram reduces endotoxin-induced fatigue. Brain Behav Immun.

[118] Felger JC, Haroon E, Miller AH (2018). What’s CRP got to do with it? Tackling the complexities of the relationship between CRP and depression. Brain Behav Immun.

[119] Horn SR, Long MM, Nelson BW, Allen NB, Fisher PA, Byrne ML (2018). Replication and reproducibility issues in the relationship between C-reactive protein and depression: A systematic review and focused meta-analysis. Brain Behav Immun.

[120] Del Giudice M, Gangestad SW (2018). Rethinking IL-6 and CRP: Why they are more than inflammatory biomarkers, and why it matters. Brain Behav Immun.

[121] Irwin MR, Cole S, Olmstead R, Breen EC, Cho JJ, Moieni M, et al. Moderators for depressed mood and systemic and transcriptional inflammatory responses: a randomized controlled trial of endotoxin. Neuropsychopharmacology. 2019;44:635–41. 10.1038/s41386-018-0259-6 PMC6333799 30391995

[122] Cho JH, Irwin MR, Eisenberger NI, Lamkin DM, Cole SW (2019). Transcriptomic predictors of inflammation-induced depressed mood. Neuropsychopharmacology.

[123] Musselman DL, Lawson DH, Gumnick JF, Manatunga AK, Penna S, Goodkin RS (2001). Paroxetine for the prevention of depression induced by high-dose interferon alfa. N. Engl J Med.

[124] Raison CL, Borisov AS, Majer M, Drake DF, Pagnoni G, Woolwine BJ (2009). Activation of central nervous system inflammatory pathways by interferon-alpha: relationship to monoamines and depression. Biol Psychiatry.

[125] Capuron L, Pagnoni G, Drake DF, Woolwine BJ, Spivey JR, Crowe RJ (2012). Dopaminergic mechanisms of reduced basal ganglia responses to hedonic reward during interferon alfa administration. Arch Gen Psychiatry.

[126] Haroon E, Felger JC, Woolwine BJ, Chen X, Parekh S, Spivey JR (2015). Age-related increases in basal ganglia glutamate are associated with TNF, reduced motivation and decreased psychomotor speed during IFN-alpha treatment: Preliminary findings. Brain Behav Immun.

[127] Dipasquale O, Cooper EA, Tibble J, Voon V, Baglio F, Baselli G (2016). Interferon-alpha acutely impairs whole-brain functional connectivity network architecture-A preliminary study. Brain Behav Immun.

[128] Dowell NG, Cooper EA, Tibble J, Voon V, Critchley HD, Cercignani M (2016). Acute changes in striatal microstructure predict the development of interferon-alpha induced fatigue. Biol Psychiatry.

[129] Dowell NG, Bouyagoub S, Tibble J, Voon V, Cercignani M, Harrison NA (2019). Interferon-alpha-induced changes in NODDI predispose to the development of fatigue. Neuroscience.

[130] Harrison NA, Brydon L, Walker C, Gray MA, Steptoe A, Critchley HD (2009). Inflammation causes mood changes through alterations in subgenual cingulate activity and mesolimbic connectivity. Biol Psychiatry.

[131] Balter LJT, Hulsken S, Aldred S, Drayson MT, Higgs S, Veldhuijzen van Zanten JJCS (2018). Low-grade inflammation decreases emotion recognition–evidence from the vaccination model of inflammation. Brain Behav Immun.

[132] Harrison NA, Brydon L, Walker C, Gray MA, Steptoe A, Dolan RJ (2009). Neural origins of human sickness in interoceptive responses to inflammation. Biol Psychiatry.

[133] Suffredini AF, Hochstein HD, McMahon FG (1999). Dose-related inflammatory effects of intravenous endotoxin in humans: evaluation of a new clinical lot of Escherichia coli O:113 endotoxin. J Infect Dis.

[134] Lasselin J, Petrovic P, Olsson MJ, Paues Goranson S, Lekander M, Jensen KB (2018). Sickness behavior is not all about the immune response: Possible roles of expectations and prediction errors in the worry of being sick. Brain Behav Immun.

[135] Lasselin J, Schedlowski M, Karshikoff B, Engler H, Lekander M, Konsman JP. Comparison of bacterial lipopolysaccharide-induced sickness behavior in rodents and humans: Relevance for symptoms of anxiety and depression. Neurosci Biobehav Rev. 2020;115:15–24. 10.1016/j.neubiorev.2020.05.001 32433924

[136] Kiers D, Koch RM, Hamers L, Gerretsen J, Thijs EJ, van Ede L (2017). Characterization of a model of systemic inflammation in humans in vivo elicited by continuous infusion of endotoxin. Sci Rep..

[137] Capuron L, Raison CL, Musselman DL, Lawson DH, Nemeroff CB, Miller AH (2003). Association of exaggerated HPA axis response to the initial injection of interferon-alpha with development of depression during interferon-alpha therapy. Am J Psychiatry.

[138] Lasselin J, Benson S, Hebebrand J, Boy K, Weskamp V, Handke A, et al. Immunological and behavioral responses to in vivo lipopolysaccharide administration in young and healthy obese and normal-weight humans. Brain Behav Immun. 2020;88:283–93. 10.1016/j.bbi.2020.05.071 32485294

[139] Evans DL, Charney DS, Lewis L, Golden RN, Gorman JM, Krishnan KR (2005). Mood disorders in the medically ill: scientific review and recommendations. Biol Psychiatry.

[140] Lange T, Dimitrov S, Born J (2010). Effects of sleep and circadian rhythm on the human immune system. Ann N. Y Acad Sci.

[141] MacDonald L, Radler M, Paolini AG, Kent S (2011). Calorie restriction attenuates LPS-induced sickness behavior and shifts hypothalamic signaling pathways to an anti-inflammatory bias. Am J Physiol Regul Integr Comp Physiol.

[142] Lasselin J, Karshikoff B, Axelsson J, Akerstedt T, Benson S, Engler H (2020). Fatigue and sleepiness responses to experimental inflammation and exploratory analysis of the effect of baseline inflammation in healthy humans. Brain Behav Immun.

[143] Andreasson A, Wicksell RK, Lodin K, Karshikoff B, Axelsson J, Lekander M. A global measure of sickness behaviour: Development of the Sickness Questionnaire. J Health Psychol. 2018;23:1452–63. 10.1177/1359105316659917 27458105

